# Topographically selective motor inhibition under threat of pain

**DOI:** 10.1097/j.pain.0000000000003301

**Published:** 2024-06-25

**Authors:** Sonia Betti, Marco Badioli, Daniela Dalbagno, Sara Garofalo, Giuseppe di Pellegrino, Francesca Starita

**Affiliations:** a Department of Psychology “Renzo Canestrari,” Center for Studies and Research in Cognitive Neuroscience, University of Bologna, Cesena, Italy; b Department of General Psychology, University of Padova, Padova, Italy

**Keywords:** Pavlovian conditioning, Fear conditioning, Threat learning, Pain anticipation, Corticospinal excitability, TMS, MEP, Skin conductance, Electrodermal activity, Noxious stimuli, Nociception, Fear of pain

## Abstract

Pain-related motor adaptations may be enacted predictively at the mere threat of pain, before pain occurrence. Yet, in humans, the neurophysiological mechanisms underlying motor adaptations in anticipation of pain remain poorly understood. We tracked the evolution of changes in corticospinal excitability (CSE) as healthy adults learned to anticipate the occurrence of lateralized, muscle-specific pain to the upper limb. Using a Pavlovian threat conditioning task, different visual stimuli predicted pain to the right or left forearm (experiment 1) or hand (experiment 2). During stimuli presentation before pain occurrence, single-pulse transcranial magnetic stimulation was applied over the left primary motor cortex to probe CSE and elicit motor evoked potentials from target right forearm and hand muscles. The correlation between participants' trait anxiety and CSE was also assessed. Results showed that threat of pain triggered corticospinal inhibition specifically in the limb where pain was expected. In addition, corticospinal inhibition was modulated relative to the threatened muscle, with threat of pain to the forearm inhibiting the forearm and hand muscles, whereas threat of pain to the hand inhibited the hand muscle only. Finally, stronger corticospinal inhibition correlated with greater trait anxiety. These results advance the mechanistic understanding of pain processes showing that pain-related motor adaptations are enacted at the mere threat of pain, as sets of anticipatory, topographically organized motor changes that are associated with the expected pain and are shaped by individual anxiety levels. Including such anticipatory motor changes into models of pain may lead to new treatments for pain-related disorders.

## Abstract 2

Supplemental Digital Content is Available in the Text.

Threat of pain triggers motor inhibition, which is topographically organized, mapped relative to the body part where pain is expected and enhanced by increasing trait anxiety.

## 1. Introduction

Pain can be considered in large part an action, rather than a perception problem, as pain motivates action to avoid harm.^12,60^ Indeed, an intimate relationship exists between pain and motor function: people move differently in response to pain, to minimize or avoid movements that may cause or exacerbate further pain.^57^ These adaptations may facilitate recovery, following acute pain; however, they may become maladaptive when maintained long term, possibly contributing to the development of chronic pain.^38^ According to the pain-adaptation model, activity of a muscle that is painful or produces a painful movement is inhibited, while that of the antagonist muscle is facilitated.^39,53^ Although the model originally attributed such changes to processes occurring at the level of the spinal cord and periphery,^53^ several studies have shown that pain also influences the excitability of the primary motor cortex (M1), with corticospinal excitability (CSE) being inhibited in response to acute experimental pain.^1,3,5,13,28,30,46,73,89–91^

Crucially, the protective function of pain does not rely just on the minimization of current tissue damage but also, and foremost, on the prevention of future instances of bodily harm. Fundamental to this process is learning.^20–24^ In fact, the evolution of the pain system may have been driven by the role of pain as a learning signal to guide the prospective reduction of harm.^79^ This view reevaluates the pain system as inherently predictive. Thus, rather than passively perceiving nociceptive inputs, the pain system may function to generate pain predictions.^12,60,79^ Considering this framework, pain-related motor adaptations may occur not only in response to pain but also be enacted predictively at the mere threat of pain. In this regard, one study found a tendency for corticospinal inhibition to occur in anticipation of a painful thermal stimulation.^25^ In addition, another study reported the anticipation of movement-related pain to be associated with corticospinal inhibition of the biceps brachii, in preparation of a painful flexion, rather than extension, movement,^61^ in line with the pain adaptation model.^39,53^ Thus, these few studies point to the possibility that the threat of pain may inhibit the corticospinal system, before any pain occurs. Nevertheless, whether and how pain drives learning by tuning the response of the motor system in anticipation of future instances of pain remains largely unexplored.

Here, we aim to advance the mechanistic understanding of pain-related motor adaptations by investigating how the motor system learns to anticipate the occurrence of pain. Given the lateralized and highly modular organization of sensorimotor circuits, with each module adapting distinct sensory inputs to motor outputs,^48^ we hypothesized that learning to anticipate a painful event may entail the acquisition of a fine-grained sensory-motor representation of the expected pain. Specifically, we hypothesized that corticospinal inhibition would mark threat of pain and that it may not only distinguish between threat and safety but also reflect the specific sensory properties of the expected pain, thus mapping where on the body it is expected to occur. To test this hypothesis, we tracked the development and evolution of changes in CSE as healthy adult participants learned to anticipate the occurrence of lateralized and muscle-specific painful events during Pavlovian threat conditioning (or fear conditioning). The latter is a reliable and widely used paradigm in which initially neutral stimuli gain motivational significance through pairing with an aversive outcome (ie, unconditioned stimulus [US]), such as a painful stimulation, thereby becoming warning cues for impending threat (ie, conditioned stimulus [CS +]) and eliciting changes in physiological response, subjective experience, and behavior, referred to as the conditioned response.^52^

Interestingly, a close interaction also exists between pain-related processes and anxiety,^19,42,68,72,88^ with anxiety being related to enhanced responses in reaction to pain. Indeed, higher trait anxiety has been related to exacerbated pain experience,^88^ to be related to decreased pain tolerance^41^ and increased pain chronicity.^33^ Thus, previous studies have found anxiety to be related to enhanced responses in reaction to pain. Nevertheless, anxiety is future oriented, characterized by exaggerated threat expectations regarding future events,^4,18^ and pathological anxiety has been related to Pavlovian threat conditioning overgeneralization and resistance to extinction.^50^ Considering this, we explored whether anxiety may be related to enhanced responses in anticipation of pain during threat conditioning. Thus, we also tested whether a correlation existed between individual differences in trait anxiety and the response of the motor system under threat of pain. Specifically, we assessed whether higher trait anxiety may be correlated to stronger inhibition of the motor system during Pavlovian threat conditioning. Understanding the relationship between anxiety and pain-related neural processes may prove informative for pain management interventions.

## 2. Methods

### 2.1. General study design and hypotheses

Two experiments on 2 independent groups of healthy individuals (experiment 1: N = 28, experiment 2: N = 30) were performed. In both experiments, participants completed a Pavlovian threat conditioning task in which 2 different colored dots (ie, conditioned stimulus [CS+]) predicted a lateralized electrocutaneous shock, whereas a third dot was used as within-subject control stimulus and never paired with shock (CS−). Specifically, the position of the shock was manipulated across the 2 experiments. In experiment 1, the CSs+ predicted the shock to the forearm, specifically CS+L predicted the shock to the left forearm, whereas CS+R to the right forearm. In experiment 2, the CSs+ predicted the shock to the hand, specifically CS+L predicted the shock to the left hand, whereas CS+R to the right hand. In both experiments, single pulse transcranial magnetic stimulation (TMS) over the left M1 was applied to probe CSE during CS presentation, before shock delivery. In this way, in both experiments, motor evoked potentials (MEPs) were acquired from the right extensor carpi radialis (ECR) forearm muscle, involved in extension and abduction of the hand at the wrist, and right first dorsal interosseous (FDI) hand muscle, involved in index finger abduction.

A reduction of MEP amplitude was hypothesized for CSs+ relative to CS−, indicating corticospinal inhibition. In addition, the MEP reduction was hypothesized to be specific for the side where the shock was expected, such that MEPs recorded from the right ECR and FDI muscles might be smaller than CS−, for CS+R specifically, which predicted right upper limb shock, rather than CS+L, which predicted left upper limb shock. Finally, if corticospinal inhibition mapped the body part, in addition to the side, where the shock was expected, MEPs reduction might be modulated also as a function of the shock position in each experiment, ie, forearm in experiment 1 or hand in experiment 2, and thus be evident in different muscle sites, namely, ECR MEPs and/or FDI MEPs, in the 2 experiments.

We also assessed skin conductance response (SCR) to have a validated physiological measure of threat conditioning. Skin conductance response is modulated by the activity of the sympathetic nervous system and is the most widely assessed conditioned response^2,6,51,62,80^ with extensive literature showing that its increase represents a robust conditioned response.^2,52^ In contrast to CSE, SCR was hypothesized to only distinguish between threat (ie, CS+) and safety (ie, CS−), regardless of the bodily laterality and location of the expected shock.^96^

Finally, trait anxiety was assessed to test the correlations between individual differences in trait anxiety and the strength of motor (ie, MEPs) and autonomic (ie, SCR) responses under threat of pain.

### 2.2. Experiment 1

#### 2.2.1. Participants

Twenty-eight healthy volunteers (14 women, aged between 20 and 35 years, mean age 24.90 ± 3.5 years) participated in the experiment. The sample size was calculated using G*Power^29^ with the following parameters: (1) analysis of variance (ANOVA): repeated measures, within factors; (2) number of groups = 1; (3) number of measurements = 3 (this refers to the main effect of stimulus type), (4) medium effect size: f = 0.25; (5) alpha = 0.05; and (6) power = 0.8.^16^ All participants were right handed, as assessed by the Edinburgh Handedness Inventory^63^ with normal or corrected-to-normal visual acuity. They were all screened for TMS exclusion criteria, to exclude any history of head trauma or head surgery, seizures and family history of epilepsy, implanted hardware, medications, neurological and medical illnesses according to safety guidelines,^74,75^ as well as any current neurological, psychiatric, and medical conditions. In addition, they had no recent history of trauma affecting the upper limbs, nor were they currently suffering from any pain or taking any analgesic medication. The study followed the American Psychological Association Ethical Principles of Psychologists and Code of Conduct and the Declaration of Helsinki and was approved by the Bioethics Committee of the University of Bologna (protocol number 224364). All participants were naive to the purposes of the experiment and gave their written informed consent for their participation.

#### 2.2.2. Experimental paradigm

##### 2.2.2.1. Pavlovian threat conditioning task

**Figure 1.**
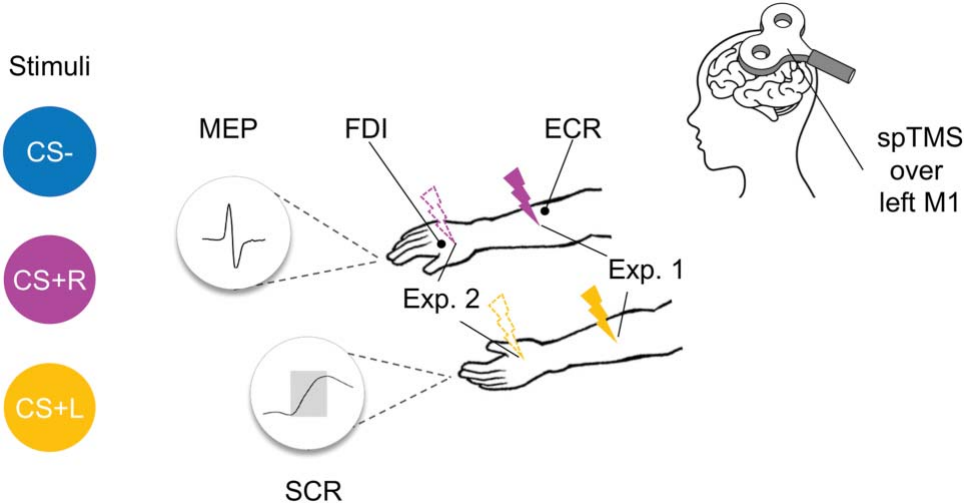
Experimental setup. Three colored dots were used as experimental stimuli: CS− (blue) was never paired with electrocutaneous shock, CS+R (pink) was associated with a right shock, and CS+L (yellow) was associated with a left shock. The shocks were delivered to the right forearm in experiment 1 (lightning, solid line) or hand in experiment 2 (lightning, dashed line). Surface electromyography (EMG) was recorded from the first dorsal interosseous (FDI) muscle of the right hand and the extensor carpi radialis (ECR) muscle of the right forearm, in both experiments. Single-pulse transcranial magnetic stimulation (spTMS) was administered to the left primary motor cortex (M1) to simultaneously elicit motor evoked potentials (MEPs) from both the FDI and ECR muscles. Skin conductance response (SCR) was acquired from 2 electrodes attached to the distal phalanges of the first and second fingers of the participant's left hand.

**Figure 2.**
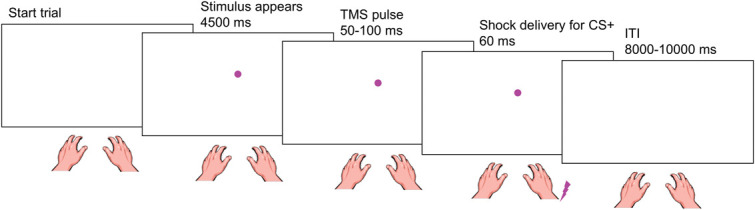
Trial structure for the Pavlovian threat conditioning task. Participants observed a colored dot (eg, pink or yellow) and learned to associate a specific color with an electrocutaneous shock (represented by a lightning), administered to the left (CS+L) or right (CS+R) forearm (experiment 1) or hand (experiment 2). Another colored dot (eg, blue) was never paired with shock (CS−). In the example, the colored dot presentation was associated with a right shock. In each trial, the colored dot appeared for 4500 ms. Afterward (following a 50-100 ms interval), spTMS was administered, and motor evoked potentials (MEPs) were recorded from the target muscles of the right hand (ie, FDI) and forearm (ie, ECR). Then, after 60 ms from the spTMS, CS+ presentation coterminated with a lateralized shock that was delivered in designated trials (duration: 2 ms), followed by an intertrial interval (ITI) of 8000 to 10,000 ms. Skin conductance was recorded continuously throughout the task. spTMS, single pulse transcranial magnetic stimulation.

During Pavlovian threat conditioning, participants learned to identify 2 colored dots as dangerous (eg, pink or yellow), representing the conditioned stimulus (ie, CS+), such that their presentation terminated with an aversive shock (ie, US) in 70% of the trials on their left (CS+L) or right (CS+R) forearm (Fig. 1). A third colored dot served as within-subject control stimulus (ie, CS−), never being paired with shock. Participants were instructed that the dots would appear one at the time on the screen and might be associated with the shock. They were also told that their task was to observe the screen and required to try to predict which color would give them the shock, while keeping their hand and arm relaxed, without executing any motor response. On each trial, the CS appeared on the screen for 4500 ms in one out of 2 positions (low, high^83^) to reduce task sameness and participants' boredom. Afterward, following an interval ranging from 50 to 100 ms, a TMS pulse was administered for MEP registration (Fig. 2), and 60 ms after the single pulse TMS, CS+ presentation coterminated with a 2-ms lateralized shock, in designated trials. Thus, CSE was probed on each trial during CS presentation, 60 ms before shock administration in designated trials, to obtain a measure reflecting shock expectancy. An intertrial interval of 8000 to 10,000 ms followed. Duration of CS and intertrial interval presentation were chosen to allow the time to measure SCR and MEP before shocks occurred, and for them to return to baseline after CS offset and/or shock delivery, before CS appearance on the next trial.^10,26,52,78,84,86^ This enabled to exclude any influence of the shock delivery per se on changes in SCR or MEPs recorded on the next trial. The first 6 acquisition trials consisted in the presentation of all types of stimuli (CS+L, CS+R, CS−) and positions (low, high) in random order; all CSs+ trials were reinforced. The remaining trials proceeded in pseudorandom order with no more than 2 consecutive stimuli of the same type in a row. The 3 CSs were presented in each position for a total of 10 repetitions each (60 trials in total). For each CS+, 14 trials (7 for each position) were associated with shock (ie, 70%), whereas the remaining 6 trials were not associated with it (30%). Color assignment to each CS was counterbalanced among participants.

#### 2.2.3. Procedure

Participants were tested individually in a single experimental session lasting approximately 2 hours. They were comfortably seated in a silent room in front of a computer screen (size: 43 inches; resolution: 1920 × 1080 pixels; refresh rate: 60 Hz), at ∼80-cm viewing distance. The stimuli consisted of 3 dots (64 pixels diameter) colored blue, pink or yellow. In all trials, stimuli appeared one at a time in one of 2 possible positions, that is, low (x = 0, y = 96) or high (x = 0, y = −192). At the beginning and at the end of the experimental session, baseline CSE was measured by acquiring MEPs while participants passively observed a fixation cross on a screen (15 repetitions per block). This was done to check for nonspecific changes in basal CSE during the experiment that could have affected the results. An interpulse interval of at least 5000 ms was adopted, thereby avoiding changes in CSE because of repeated TMS pulses.^11^ Before the threat conditioning task, the shock electrodes were attached to the participants' forearms, and shock intensity was calibrated. At the end of the experimental session, participants rated the valence of each CS and the CS-US expectancy and contingency (see the supplemental information, http://links.lww.com/PAIN/C76) and filled out the State-Trait Anxiety Inventory questionnaire (STAI^65,82^). At the end of the whole experiment, they were debriefed about the experimental hypotheses. A computer running the OpenSesame software^54^ connected with a NI USB-6281 device (National Instruments, Austin, TX), and a BIOPAC MP-150 System (BIOPAC Systems, Goleta, CA) controlled the flow of the task and data recordings.

#### 2.2.4. Stimulations and recordings

##### 2.2.4.1. Electrocutaneous stimulation

The US consisted of a 2-ms electrocutaneous stimulation generated by a Digitimer Stimulator (Model DS7A; Digitimer Ltd, Hertfordshire, United Kingdom) and delivered to the participants' left and right forearms (for CS+L and CS+R, respectively), proximal to the ECR muscle (Fig. 1), through pregelled Ag/AgCl snapped electrodes (Friendship Medical, SEAg-S-15000/15x20). At the beginning of the calibration procedure, participants were informed that the aim of the calibration procedure was to reach “a stimulation intensity at the threshold between highly unpleasant and painful, to the point that if they knew it was coming, they would prefer not to experience it.” We also informed them that we would start the calibration at a low intensity, to gradually increase it to the instructed level of perception. We then proceeded with the calibration, using a standard staircase procedure with increasing intensities, starting from 0 mA and increasing in steps of 5 mA. When the participant reported that the stimulation intensity level was reached, we asked them to rate the experience of that stimulation on a Likert scale ranging from 0 (no sensation) to 10 (painful). If a score of 8 was reported, that intensity was selected for the rest of the experiment. If a lower score was reported, we asked the participant if they were comfortable to have the intensity increased, and eventually proceeded with the delivery of the following stimulation/s until a score of 8 was reached. Alternatively, if the participant was not comfortable with the increase in stimulation, we stopped at that intensity, in agreement with the International Association for the Study of Pain indications that “in any pain research, stimuli should never exceed a subject's tolerance limit.” This procedure resulted in a stimulation intensity of (mean ± SD) 31.30 ± 20.78 and 32.58 ± 18.48 mA for the left and right forearms, respectively. In addition, a self-reported level of rating of 8.02 ± 0.09 and 8.00 ± 0.02 was reported for the left and right forearms, respectively.

Electrocutaneous stimulation was used as the painful stimulus because it has long been used in the literature that studies the neurophysiological bases of pain, pain anticipation, fear of pain, and Pavlovian threat conditioning, and it is known to produce specific transient modulations of psychophysiological responses that can be assessed on a trial-by-trial basis.^52,58^ In addition, unlike some other devices, such as CO_2_ laser, electrocutaneous stimulation allows repeated stimulation at the same site without the risk of tissue damage.^17^

##### 2.2.4.2. Transcranial magnetic stimulation

Single-pulse TMS was administered using a 70-mm figure-of-eight coil connected to a Magstim Rapid2 stimulator (Magstim, Co, Whitland, United Kingdom). Standard procedures of the MEP literature (see eg, Refs. 36 and 77 for methodological guidelines^7–9^) were used for TMS application. Pulses were delivered to the left M1 of the participant, in correspondence with the target muscle representation (Fig. 1). The coil was placed on the head at a 45-degree angle relative to the interhemispheric fissure, with the handle pointing laterally and caudally.^10,59^ The optimal scalp position, which is the best position for the coil on the scalp at which the lower intensity of stimulation elicits the largest MEP of both the right hand FDI and right forearm ECR muscles, was determined by moving the coil in approximately 0.5-cm steps around the presumed hand motor area. Once found, the optimal scalp position was then marked on a tight-fitting cap worn by the participants, ensuring a correct coil placement throughout the experiment. For each participant, the resting motor threshold (rMT) was then determined by finding the lowest stimulation intensity inducing MEPs with at least ≥ 50 µV peak-to-peak amplitude in a relaxed muscle in 5 out of 10 trials.^76^ During the experiment, the intensity of TMS stimulation was then set at 120% of the individual rMT. Resting motor threshold ranged from 29% to 81% (mean = 57.9%, SD = 12.0) of the maximum stimulator output. Motor evoked potentials recorded during the experiment were then preprocessed and analyzed as described in the dependent variables section.

##### 2.2.4.3. Electromyography recording

Surface electromyography (EMG) activity was recorded simultaneously from the right FDI hand muscle, involved in index finger abduction, and right ECR forearm muscle, involved in extension and abduction of the hand at the wrist, through 2 pairs of Ag/AgCl electrodes (BIOPAC EL501, BIOPAC Systems) placed in a belly‐tendon montage connected to an EMG100C module of the BIOPAC MP-150 System. After skin preparation, a small amount of isotonic hyposaturated conductant gel (Lectron III Gel; NEUROSPEC) was added to the electrodes, which were placed and fixed on the target positions. The active electrode was placed over the muscle belly, determined by palpation during maximum voluntary contraction, and the reference and ground electrodes were placed over the proximal interphalangeal juncture and the radial styloid process for the FDI muscle and over the ulnar styloid process and the lateral epicondyle for the ECR muscle.

##### 2.2.4.4. Skin conductance response recording

Skin conductance was recorded throughout each phase at 5000 Hz, with a 10 Hz lowpass filter, from 2 Ag/AgCl electrodes (TSD203; BIOPAC Systems) filled with isotonic hyposaturated conductant gel (GEL101 model; BIOPAC System) attached to the distal phalanges of the first and second fingers of participants' nondominant hands, connected to an EDA100C module of the BIOPAC MP-150 System (BIOPAC Systems).

### 2.3. Dependent variables

#### 2.3.1. Motor evoked potential

**Figure 3.**
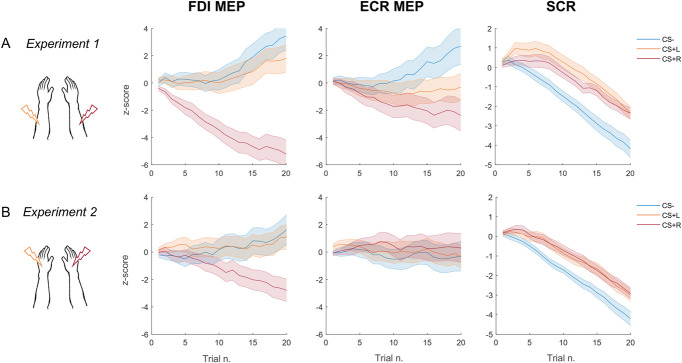
Trial-by-trial means and standard errors (shaded area) of cumulative *z*-scores for FDI and ECR motor evoked potentials (MEPs) (left and middle graph, respectively) and skin conductance response (SCR) (right graph) for CS− (blue), CS+L (orange), and CS+R (dark red) stimuli in experiment 1 (panel A) and experiment 2 (panel B) throughout the 20 trials of Pavlovian threat conditioning. ECR, extensor carpi radialis; FDI, first dorsal interosseous.

Electromyography data were preprocessed offline in MATLAB using custom-made scripts. First, epochs from the time of TMS pulse to 60 ms after it were extracted from the continuous data for MEP identification and analysis, and epochs from −100 ms to the time of TMS pulse were extracted to identify any muscular preactivation, before TMS delivery. For each epoch, the min and max MATLAB functions were then used to identify the minimum and maximum peak in the EMG signal. The signal was then visually inspected to ensure the correctness of the automatic procedure, such that manual correction could be performed in case of any peak misidentification. Nevertheless, peak misidentification never occurred in the current data set, and manual correction was never applied. Muscular preactivations were defined as trials in which peaks of EMG activity in the 100-ms window preceding the TMS pulse exceeded 2 SD from the mean background EMG activity. Motor evoked potentials preceded by such preactivation were discarded to prevent contamination of the MEP measurements (a total of 0.84% and 1.34% of MEPs were excluded for FDI and ECR, respectively). This enabled to exclude any influence of participants' movement or movement that may have been evoked by shock delivery to influence MEPs recorded on the next trial. For remaining trials, individual peak‐to‐peak MEP amplitudes (mV) for the FDI and ECR muscles were calculated and considered as a proxy for CSE during threat conditioning. Then, MEPs' whose amplitude exceeded ±2 SD of the mean amplitude for each experimental condition were excluded as outliers (CS−: 1.30% and 1.11%; CS+L: 1.60% and 1.23%; CS+R: 1.42% and 1.17% for FDI and ECR, respectively). To control for interindividual variability in MEP amplitudes, the raw MEP amplitudes were z‐transformed using the average and standard deviation calculated on all CS conditions, separately for each participant and each muscle. The MEP data for one subject could not be analyzed because of technical issues during data registration. To characterize the trend of MEP amplitude modulations during threat conditioning, we calculated the cumulative sum of MEP *z*-score for each CS (regardless of its position, ie, high, low) throughout the 20 trials that constituted the threat conditioning task (see Fig. 3A and Supplemental information, http://links.lww.com/PAIN/C76).

#### 2.3.2. Skin conductance response

The digitalized electrodermal activity signal was processed using Autonomate 2.8^35^ running in MATLAB (The Mathworks) to obtain trough-to-peak SCR values. The SCR was considered valid if the trough-to-peak response occurred between 500 and 4500 ms following the stimulus onset, lasted for a maximum of 5000 ms, and was greater than 0.02 µS. Raw SCR data were z-transformed, and values exceeded ± 2 SD of the sample mean were excluded as outliers (CS−: 1.01%; CS+L: 1.25%; CS+R: 1.85%). As for the MEPs, to characterize the trend of SCR modulations during threat conditioning, we calculated the cumulative sum of SCR *z*-score for each CS (regardless of its position, ie, high, low) throughout the 20 trials that constituted the conditioning procedure (Fig. 3A).

#### 2.3.3. State-Trait Anxiety Inventory

At the end of the experimental session, participants filled the Italian version of the trait scale of the STAI form Y-2.^65,82^ The questionnaire is composed of 20 items that investigate how the subject habitually feels (eg, “I feel nervous and restless”), higher scores indicate greater anxiety.

#### 2.3.4. Statistical analysis

Analyses were performed with JASP 0.16.3 (JASP Team, 2022). Repeated-measures ANOVA (rmANOVA) were used to investigate differences between more than 2 conditions. For SCR data, CS (CS−, CS+L, CS+R) was considered as a within‐subject factor in the analysis, whereas for MEP data, the additional factor muscle (FDI, ECR) was also considered. For both SCR and MEP data, planned contrasts were adopted to test for differences among CSs due to the threat conditioning (ie, CS− vs CS+L, CS− vs CS+R, CS+L vs CS+R). Degrees of freedom and *P*-values were Greenhouse–Geisser corrected, whenever a violation of the sphericity assumption occurred. To test for any basal change in CSE during the experiment, the raw MEPs acquired during the initial and final baseline blocks were compared through paired *t*‐tests. Partial eta-squared ($\eta_{p}^{2}$) and 90% confidence intervals (CIs) were computed as estimates of effect sizes for the ANOVAs' main effects and interactions. Pearson correlations (one-tailed) were computed to assess the relationship between trait anxiety and our motor and autonomic measures. For this purpose, we correlated the total cumulative sum of MEP and SCR variables (*z*-scores) with the score of the STAI-trait questionnaire. To increase power, data of both experiments 1 and 2 were used for the correlations. A statistical significance threshold of *P* < 0.05 was adopted for all analyses.

### 2.4. Experiment 2

#### 2.4.1. Participants

Thirty healthy right-handed volunteers (15 women, aged between 20 and 30 years, mean age 22.83 ± 2.29 years) with the same characteristics as those participating in experiment 1 were tested. No participant who took part in experiment 1 was recruited for experiment 2, to prevent any effect of having previously participated in a similar threat conditioning experiment from influencing the results of experiment 2.

#### 2.4.2. Experimental paradigm and data recordings

The same apparatus, stimuli, and procedures were adopted as for experiment 1, but during threat learning, electrocutaneous stimulation was administered to the participants' left and right hand (for CS+L and CS+R, respectively), proximal to the FDI muscle (Fig. 1).

As for experiment 1, the shock intensity calibration resulted in the following intensities (left hand: M = 33.30 mA, SD = 18.7; right hand: M = 32.17 mA, SD = 16.09). Participants rated the shock on a scale ranging from 0 to 10, from no sensation to painful (left hand: M = 7.96, SD = 0.35; right hand: M = 8.05, SD = 0.33). The electrodermal activity was analyzed having raw SCR data z-transformed, and values that exceeded ± 2 SD of the sample mean were excluded as outliers (CS−: 1.11%; CS+L: 2.00%; CS+R: 1.44%). As regards MEP recordings, rMT ranged from 53% to 93% (mean = 70.13%, SD = 9.93) of the maximum stimulator output. Trials in which peaks of EMG activity in the 100-ms window preceding the TMS pulse exceeded 2 SD from the mean background EMG activity were discarded to prevent contamination of the MEP measurements (a total of 2.09% and 1.87% of MEPs were excluded for FDI and ECR, respectively). In addition, values exceeded ± 2SD of the mean amplitude for each experimental condition were excluded as outliers (CS−: 1.22% and 1.28%; CS+L: 1.17% and 0.89%; CS+R: 1.44% and 1.22% for FDI and ECR, respectively).

#### 2.4.3. Data analysis

All data were analyzed as described in experiment 1.

## 3. Results

### 3.1. Experiment 1

#### 3.1.1. Trial by trial acquisition of conditioned corticospinal response and skin conductance response

Figure 3A shows cumulative sum of MEP and SCR amplitudes (*z*-scores) throughout the 20 trials of threat conditioning that was computed to characterize the development of conditioned responses over experimental trials in experiment 1. Regarding MEP, the graphs show a different pattern that characterizes MEPs responses for the FDI and ECR muscles among CSs. For the FDI muscle, MEPs show smaller amplitudes throughout the conditioning trials when CS+R was presented as compared with CS+L and CS−, with the difference between MEP amplitude between CS+R and CS+L and CS− increasing over the course of conditioning. For the ECR muscle, MEPs show smaller amplitudes throughout the conditioning trials when CS+R was presented as compared with CS−, with the difference between the 2 stimuli increasing over the course of conditioning. In contrast, a flat trend of response appears for CS+L MEPs. For SCR, a higher cumulative trough-to-peak amplitude characterizes responses to CSs+ presentation compared with CS−, regardless of the side of shock, and net of an overall decrease in SCR over experimental trials indicative of habituation.

#### 3.1.2. Corticospinal excitability

**Figure 4.**
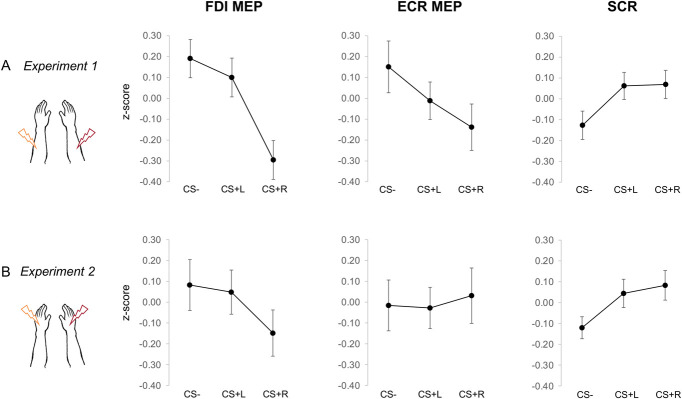
Corticospinal excitability and skin conductance responses during threat conditioning. Group estimate marginal means and 95% confidence interval (CI) of motor evoked potential (MEP) amplitudes (*z*-score) for the FDI and ECR muscles and SCR (*z*-score) as a function of the CS type in experiment 1 (A) and experiment 2 (B). SCR, skin conductance response. ECR, extensor carpi radialis; FDI, first dorsal interosseous.

A rmANOVA was performed on mean MEP amplitude (*z*-scores) with muscle (FDI, ECR) and CS (CS−, CS+L, CS+R) as within‐subject factors. We observed a main effect of CS (F_2,52_ = 14.52, *P* < 0.001, $\eta_{p}^{2}$ = 0.36, 90% CI: [0.17-0.48]) and an interaction between muscle and CS (F_2,52_ = 5.08, *P* = 0.010, $\eta_{P}^{2}$ = 0.16, 90% CI: [0.02-0.29]). No main effect of muscle emerged (F_1,26_ = 1.00, *P* = 0.326, $\eta_{P}^{2}$ = 0.04, 90% CI: [0-0.20]). Planned comparisons showed that the CS+R MEP were significantly reduced compared with the CS− for both FDI (t_84.74_ = −5.69, *P* < 0.001) and ECR (t_84.74_ = −3.39, *P* = 0.001) muscles and compared with the CS+L, but only for the FDI muscle (t_84.74_ = 4.63, *P* < 0.001; see Fig. 4A). In contrast, there was no significant difference in MEP amplitude between CS+L and CS− neither for FDI (t_84.74_ = −1.06, *P* = 0.291) or ECR muscle (t_84.74_ = −1.48, *P* = 0.060). These results point to a side-congruent inhibitory effect, with a significant reduction of right MEP amplitudes in anticipation of side-congruent shock.

Note that no significant differences emerged when comparing raw MEPs recorded during the initial and final baseline blocks, either for FDI and ECR muscles (t_26_ = 0.14, *P* = 0.887 and t_26_ = −0.77, *P* = 0.448, respectively). This indicates that TMS, the task, or the shocks per se did not induce general, long-lasting changes in motor excitability during the experiment.

#### 3.1.3. Skin conductance response

A rmANOVA was performed with CS (CS−, CS+L, CS+R) as within‐subject factor to analyze SCR data. We observed a main effect of CS (F_2,46_ = 7.87, *P* = 0.001, $\eta_{P}^{2}$ = 0.26, 90% CI: [0.07-0.39]). Planned comparisons showed that SCR was significantly higher for both CS+L and CS+R than for CS− (t_46_ = 3.38, *P* = 0.001 and t_46_ = 3.49, *P* = 0.001, respectively), and it did not differ between CSs+ (t_46_ = 0.11, *P* = 0.911; Fig. 4A). Participants showed higher arousal to the CSs+ than for the CS−, regardless of the laterality of the expected shock.

### 3.2. Experiment 2

To test whether CSE, and thus MEP responses, recorded at the different muscle sites (FDI or ECR) would be modulated by the shock position, we conducted a second experiment. The procedures were analogous to those of experiment 1, except for the site of electrocutaneous stimulation delivery. In experiment 1, the electrocutaneous stimulation was administered to the participants' left and right forearm, in the proximity of the ECR muscle, whereas in experiment 2, the electrocutaneous stimulation was administered to the participants' left and right hands (for CS+L and CS+R, respectively), in the proximity of the FDI muscle.

#### 3.2.1. Trial by trial acquisition of conditioned corticospinal excitability response and skin conductance response

Figure 3B shows cumulative sum of MEP and SCR amplitudes (*z*-scores) throughout the 20 trials of threat conditioning that was computed to characterize the development of conditioned responses over experimental trials in experiment 2. Regarding MEP, the graphs show a different pattern that characterizes MEPs responses for the FDI and ECR muscles among CSs. For the FDI muscle, MEPs show smaller amplitudes throughout the conditioning trials when CS+R was presented as compared with CS+L and CS−, with the difference between MEP amplitude between CS+R and CS+L and CS− increasing over the course of conditioning. For the ECR muscle, MEP amplitudes do not appear to differentiate among the 3 CSs. For SCR, a higher cumulative trough-to-peak amplitude characterizes responses to CSs+ presentation compared with CS−, regardless of the side of shock, and net of an overall decrease in SCR over experimental trials indicative of habituation.

#### 3.2.2. Corticospinal excitability

A rmANOVA was performed with muscle (FDI, ECR) and CS (CS−, CS+L, CS+R) as within‐subject factors to analyze MEP data during the Pavlovian threat conditioning phase. We observed an interaction between muscle and CS (F_2,58_ = 5.75, *P* = 0.005, $\eta_{P}^{2}$ = 0.17, 90% CI: [0.03-0.29]). No main effect emerged (all F < 0.61, *P* > 0.546). Planned comparisons showed that the CS+R MEP were significantly reduced compared with the CS− for FDI (t_87.79_ = −2.36, *P* = 0.020) but not the ECR (t_87.79_ = 0.48, *P* = 0.63) muscle (Fig. 4B). In addition, there was no significant difference in MEP amplitude between CS+L and CS−, neither for FDI (t_87.79_ = −0.35, *P* = 0.724) or ECR muscle (t_87.79_ = 0.13, *P* = 0.90). These results point to a side-congruent inhibition of CSE for the FDI muscle, with a significant reduction of MEP amplitude in anticipation of side-congruent shock.

Note that no significant differences emerged when comparing raw MEPs recorded during the initial and final baseline blocks, neither for FDI and ECR muscles (t_28_ = 0.79, *P* = 0.438 and t_28_ = −0.47, *P* = 0.641, respectively). This indicates that TMS, the task, or the shocks per se did not induce general, long-lasting changes in motor excitability during the experiment.

#### 3.2.3. Skin conductance response

A rmANOVA was performed with CS (CS−, CS+L, CS+R) as within‐subject factor to analyze SCR data. We observed a main effect of CS (F_2,50_ = 7.98, *P* < 0.001, $\eta_{P}^{2}$ = 0.242, 90% CI: [0.07-0.36]). Planned comparisons showed that SCR was significantly higher for both CS+L and CS+R than for CS− (t_50_ = 3.05, *P* = 0.004 and t_50_ = 3.76, *P* < 0.001, respectively) but did not differ between CSs+ (t_50_ = 0.71, *P* = 0.479; Fig. 4B). Participants showed higher arousal to the CSs+ than to the CS−, regardless of the laterality of the expected shock.

### 3.3. Correlations between motor evoked potentials and skin conductance response and trait anxiety

To test the correlation between individual differences in trait anxiety and the strength of motor and autonomic responses under the threat of pain, we used the MEPs' and SCR's cumulative sums (*z*-scores) as an overall quantification of the accumulated information regarding the pain-predictive value of the CSs at the end of threat conditioning. Thus, Pearson correlations were computed between participants' STAI trait scores and MEPs' and SCR's cumulative sums (*z*-scores) to CS+ and CS−, combining the data of the 2 experiments to increase statistical power. For MEP correlations, MEPs recorded from the FDI muscle were used as it discriminated between CS+ and CS− in both experiments 1 and 2, unlike the ECR muscle.

**Figure 5.**
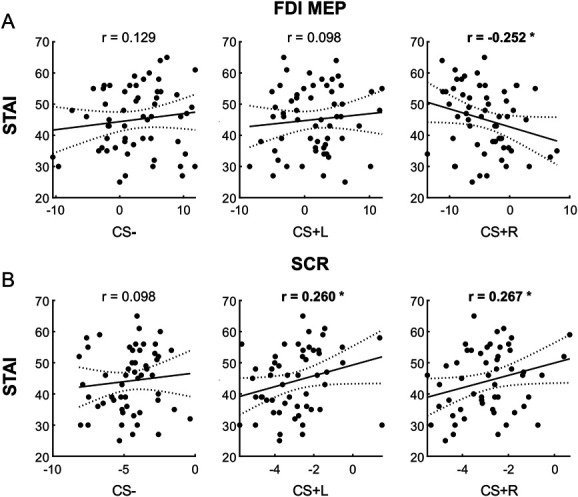
Correlations between STAI trait anxiety scores and FDI motor evoked potential (MEP) (A) and skin conductance response (SCR) (B) responses to Pavlovian threat conditioning. Dotted lines represent 95% confidence interval (CI). STAI, State-Trait Anxiety Inventory.

Results show a significant negative correlation between STAI scores and FDI MEPs for CS+R (*r* = −0.252, *P* = 0.029, CI: [−1 to −0.03]; Fig. 5A). No other significant correlations between STAI and MEP emerged (CS−: *r* = 0.129, *P* = 0.830, CI: [−1 to 0.34]; CS+L: *r* = 0.098, *P* = 0.765, CI: [−1 to 0.31]; Fig. 4A). Thus, higher levels of trait anxiety were associated with lower CSE in the anticipation of the right, but not left, shock. Regarding SCR (Fig. 5B), a positive correlation emerged between STAI trait scores and SCR for both CS+L (*r* = 0.260, *P* = 0.030, CI: [0.03-1]) and CS+R (*r* = 0.267, *P* = 0.027, CI: [0.04-1]), whereas no significant correlation emerged for CS− (*r* = 0.098, *P* = 0.243, CI: [−0.13 to 1]). This suggests that higher levels of trait anxiety were associated with higher SCR in anticipation of a shock, regardless of shock laterality.

## 4. Discussion

This work advances the neurophysiological and mechanistic understanding of the changes in motor control under the threat of pain and their relation to individual differences in anxiety, which closely interacts with pain related processes.^19,33,41,42,68,72,88^ This study aimed to investigate how the motor system learns to anticipate the occurrence of pain. To this end, CSE was probed during Pavlovian threat conditioning, by eliciting MEPs shortly before the time of shock delivery. This procedure enabled us to track the development and evolution of changes in CSE on a trial-by-trial basis, as learning progressed. In addition, we assessed the level of participants trait anxiety, with the State-Trait Anxiety Inventory (STAI form Y-2^65,82^), and tested the correlation between participants' trait anxiety and the cumulative changes in CSE resulting at the end of threat conditioning. Our results show that even when pain occurs unconditional to any overt motor response, learning to anticipate bodily harm entails the acquisition of a set of topographically organized sensorimotor contingencies associated with the expected pain. Such results highlight the crucial role played by cognition in the enactment of pain-related motor adaptations, showing that the mere threat of pain is sufficient to precisely shape motor system activity, even in the absence of any actual pain.

We show that the threat of pain results in corticospinal inhibition, which occurs before the actual occurrence of any harm and is mapped onto the body part where pain is expected. In details, during Pavlovian threat conditioning, CSE was reliably conditioned to discriminate between threat and safety, as the presentation of a shock predicting stimulus, ie, CS+, triggered corticospinal inhibition immediately before shock occurrence. Indeed, a reduction in MEP amplitude recorded from the right arm and hand was found under threat of pain to the right forearm (experiment 1) or hand (experiment 2), ie, in the presence of CS+R but not CS−. This result was found for the right FDI muscle in both experiments and for the right ECR muscle in experiment 1 but not 2. In addition, cumulative trial-by-trial responses showed that the difference in MEP amplitude between CS+R and CS− appeared to increase over the course of experimental trials, indicating an increasing discrimination of the 2 stimuli as learning progressed. In contrast, when pain was expected on the left forearm (experiment 1) or hand (experiment 2), ie, in the presence of CS+L, no significant difference from CS− was found in MEP amplitude, in either muscle or experiment.

Overall, MEP results indicate a side-congruent inhibition of CSE, specific for the upper limb on which the shock is expected to occur. Crucially, they suggest that conditioned inhibition of CSE develops beyond coarse laterality, by mapping also the body part where the shock is expected. Indeed, moving the site of shock delivery from a more proximal to a more distal upper limb position, ie, from the forearm to the hand, produced a corresponding shift in the pattern of corticospinal inhibition. Although threat of pain to the forearm resulted in corticospinal inhibition for both the forearm and hand muscles, threat of pain to the hand resulted in a specific inhibition for the hand muscle. Such nonuniform effects of CSE across distal and proximal muscles of the upper limb have been observed before in response to pain and are thought to be part of a complex protective reflex mechanism in the upper limb of humans.^43,45^ Indeed, they resemble nonuniform effects also shown in the limb withdrawal reflex, which appears to be flexibly modulated by the site of pain delivery.^14,81^ Here, we extend this evidence showing that this mechanism may be predictively recruited at the mere threat of pain, before any pain occurs. This evidence also extends previous results from functional magnetic resonance imaging and electroencephalography that showed the activation of motor areas when learning to expect a painful outcome,^31,67,85,95,96^ revealing that such learning entails the acquisition of a fine-grained, topographically organized motor representation of expected pain, even when this occurs unconditional to any motor response.

Regarding the relation between CSE under threat of pain and individual differences in trait anxiety, we found that greater trait anxiety correlated with greater corticospinal inhibition, once again, specific for the arm where the shock was expected. Previous studies found trait anxiety to be related to psychological responses in reaction to pain. For example, higher trait anxiety exacerbates pain experience and is related to decreased pain tolerance.^41,88^ Here, we add to this evidence, showing a positive correlation between trait anxiety and a neurophysiological response in anticipation of pain, before the actual occurrence of any painful event. Given that anxiety is characterized by apprehensive expectations about future events,^4,18^ enhanced motor inhibition under the threat of pain in more anxious individuals may be a neurological marker of exaggerated pain expectations about the impending shock and in turn influence the psychological response to pain. Further exploring the relationship between trait anxiety, neurophysiological responses under the threat of pain, and subjective responses in reaction to pain may be important to advance the understanding of the complex, multidimensional, pain processes and to improve pain management interventions.

The results are also relevant for the Pavlovian conditioning literature. In fact, in humans, threat conditioning is rarely studied through the lens of the motor system,^6^ and changes in motor responses are just deemed as one of its indirect consequences.^22,27,40,71^ The topographically organized modulation of a conditioned response, here found for CSE, has never been reported in studies using more commonly assessed conditioned responses during Pavlovian threat conditioning, either motor (eg, startle eye-blink response, postural freezing) or autonomic (eg, SCR, pupil, bradycardia).^62,92^ Given this peculiarity, the conditioned corticospinal response has the potential to provide novel and unique information regarding the development and maintenance of adaptive and maladaptive threat learning,^49^ as suggested by the positive correlation we found between participants' level of anxiety and strength of the conditioned corticospinal response. In contrast to the selectively side-congruent response of the motor system, response of the autonomic system, assessed through SCR, did not show any evidence of side congruency. In both experiments and in line with previous evidence,^96^ participants showed higher SCR to the CSs+ than to the CS−, regardless of the laterality of shock. This absence of side congruency was corroborated by correlation analyses, which showed a positive correlation between trait anxiety and SCR, both for CS+R and CS+L, indicating that greater trait anxiety was related to greater SCR, regardless of CS-US laterality. The different modulation of CSE and SCR supports the notion that Pavlovian threat conditioning involves multiple systems, responsible for different classes of conditioned responses. Although some responses (also known as consummatory responses) are outcome specific, encoding the sensory properties of the outcome, such as its bodily location, other responses are more general, encoding only its motivational value, such as its painful (or not) nature.^23,37,69,70,96^ In this regard, our results further corroborate the preparatory nature of the SCR, which was not modulated by shock laterality, and, crucially, advance the understanding of consummatory responses, expanding their neural correlates^96^ to include the cortical motor system because corticospinal inhibition was side congruent to the expected shock and modulated by its position on the hand or forearm. The somatotopic-like modulation of the conditioned corticospinal inhibition suggests that consummatory responses encode a significantly more detailed representation of the sensory properties of the outcome than previously known in humans.

Potential limitations of this study and directions for future studies should also be discussed. The side-congruent motor inhibition was found by probing CSE from the left M1, recording MEPs from muscles of the right upper limb. Although we hypothesize a similar inhibition for the left M1, further studies should be conducted to experimentally test whether this is the case. In addition, the potential impact of the lack of active motor task should be considered when reflecting on the processes that this study has tapped into. To enable MEPs recording, participants were asked to not move and relax their muscles, although they anticipated the arrival of the somatosensory painful stimulus. These task demands may resemble those of a doctor asking the patient to relax their arm before vaccine injection. In these circumstances, motor inhibition may prevent movements that may otherwise exacerbate an unavoidable pain. Interestingly, the anticipation of movement-related pain has similarly been shown to inhibit the excitability of painful agonist muscles,^61^ suggesting that motor inhibition may be a more general process underlying pain anticipation, independently from movement preparation. Nevertheless, whether motor inhibition also occurs in preparation of an action aimed at reducing pain warrants further investigation. Finally, along with trait anxiety, individual differences in fear of pain,^56^ pain hypervigilance,^55^ and pain catastrophizing^87^ should be assessed by future studies. Given their relevance in the development and maintenance of pain-related disability,^94^ their relationship with CSE under the threat of pain may advance the understanding of the development and maintenance of pathological pain responses. In addition, future studies may dig deeper into understanding the functional role of corticospinal inhibition in relation to subjective pain perception.^13^ For example, the assessment of trial-by-trial pain ratings following shock delivery could be integrated in the current paradigm, to test whether the strength of corticospinal inhibition under the threat of pain may enhance or reduce subsequent pain perception.

The corticospinal inhibition here found under the threat of pain has crucial implications for the understanding of pain processes, also in clinical contexts, as aberrant threat learning has been hypothesized to contribute to the transition from acute to chronic pain.^93^ Indeed, the protective function of pain relies on motor responses more than perception^44,60^ and an emerging literature is growing on the modulatory role that motor response have on pain perception and management.^15,32,34,47,64,66^ In this regard, substantial evidence shows corticospinal inhibition in response to experimentally induced pain.^13^ In addition, such motor inhibition has been related to pain severity, such that, for short-lasting pain, greater inhibition relates to lower pain, whereas for long-lasting pain, it relates to higher pain.^13^ This evidence is in line with the idea that following acute pain, motor adaptations may be adaptive to prevent further injury; however, they may become maladaptive when maintained long term, contributing to pain recurrence or persistence,^38^ possibly, because enduring motor system inhibition may interfere with physical therapy and motor control rehabilitation, which would otherwise contribute to pain resolution. This study suggests that such complex interactions between pain and the motor system may be enacted as soon as individuals think that pain may occur or re-occur, even in absence of ongoing pain or injury, and especially in highly anxious individuals. In addition, the present study corroborates the idea that resolution of pain is unlikely to restore motor function, as motor adaptations do not require concurrent pain.^38^ Thus, along physical therapy, interventions that directly target plasticity of the corticospinal system may be required together with restructuring of unhelpful cognitions such as catastrophizing pain expectations and anxious thoughts.

## Conflict of interest statement

The authors have no conflicts of interest to declare.

The data that support the findings of this study are available from the authors F.S. and S.B.

## Appendix A. Supplemental digital content

Supplemental digital content associated with this article can be found online at http://links.lww.com/PAIN/C76.

## Supplementary Material
