# Supplementary material for: Topographically selective motor inhibition under threat of pain
Source: Pain. 2024 Jun 25;165(12):2851–62. doi: 10.1097/j.pain.0000000000003301 (PMC11562763; doi:10.1097/j.pain.0000000000003301)
Supplement: Supplementary file 1 [file jop-165-2851-s001.pdf]

Supplemental information

Material and methods

Self-report measures. In each experiment, to obtain an explicit measure of the subjective experience of CSs, at the end of threat learning we asked participants to rate the valence of each stimulus as well as the expectancy and contingency of each colored dot and the potential lateralized shock delivery. Participants were requested to respond using a 11-point Likert scale (ranging from 0 to 10) to three questions, each referred to all the three stimuli presented in a random order: i) "The feeling I had when the dot was this color was" (Valence; ranging from 'unpleasant' to 'pleasant'); ii) "When I saw this dot, I expected to get the shock on my left or right arm/hand" (Expectancy; from 'never' to 'always'); iii) "When the dot was this color, it gave me the shock on my left or right arm/hand" (Contingency; from 'never' to 'always').

Cumulative sums of conditioned corticospinal response and SCR. For each CS, the cumulative sum of MEP and SCR z-scores throughout the 20 trials that constituted the learning procedure was computed to quantify trial-by-trial accumulation of information regarding the value of the stimulus (see also Figure 3). The score of the last trial of cumulative sums for each CS was used in the statistical analyses.

Results

Experiment 1

Self-report measures. We conducted planned contrasts to test whether explicit subjective ratings of stimulus valence were modulated by the association (or absence thereof) of CSs with the lateralized US. Participants rated the CS+L and the CS+R as less pleasant than the CS- ($t_{54} = –5.57$, $p < 0.001$ and $t_{54} = –5.83$, $p < 0.001$, respectively; see Figure S1A), whereas ratings for CS+L and CS+R did not differ ($t_{54} = –0.27$, $p = 0.792$).

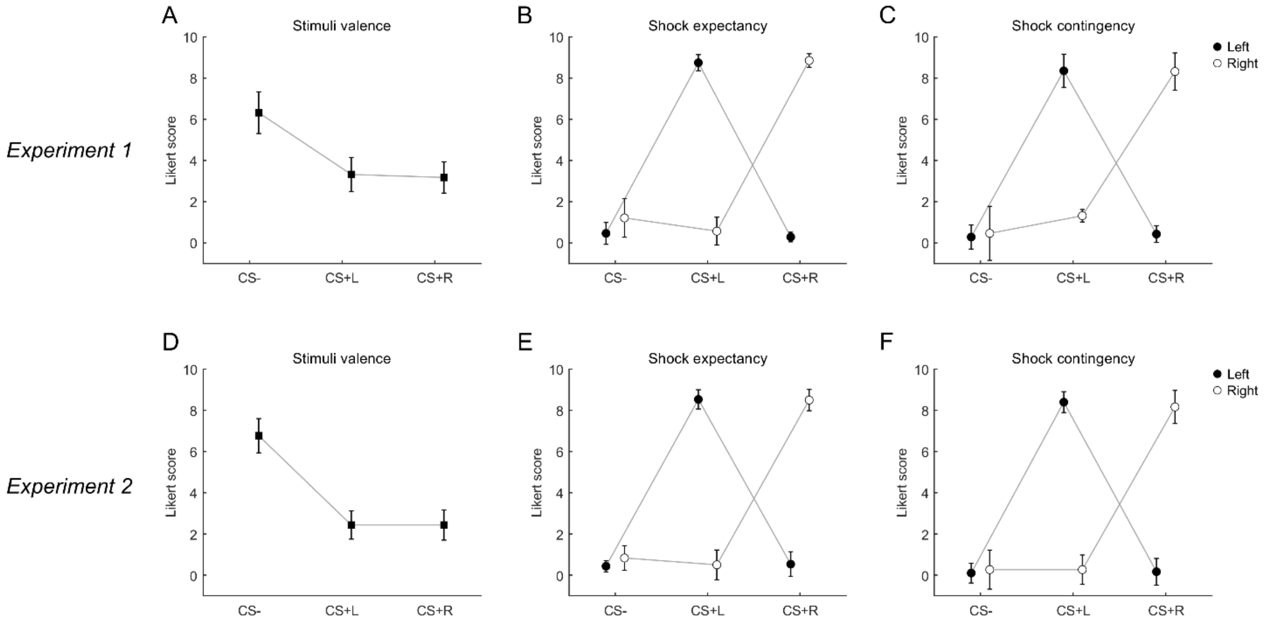

Figure S1. Subjective ratings. Likert-scale ratings for stimuli valence (A, D), CS-US expectancy (B, E), and CS-US contingency (C, F) for Experiment 1 and 2, respectively. The error bars represent 95% CI.

Regarding the expectation associated with each CS to receive a left or right US, planned contrasts on explicit subjective ratings revealed that participants rated the expectation of receiving a left arm shock when presented with a CS+L higher compared to both the CS+R and CS- ($t_{97.973} = 27.30$, $p < 0.001$). A similar pattern emerged when participants rated the expectation of receiving a right arm shock following the CS+R presentation compared to both the CS+L and CS- ($t_{97.973} = 25.96$, $p < 0.001$; Figure S1B).

Similarly, planned contrasts on the reported contingency of each CS with a left or right US showed that higher scores were given when participants were to assess that they received a left arm shock when presented with a CS+L higher compared to both the CS+R and CS- ($t_{106.118} = 24.44$, $p < 0.001$). A similar pattern emerged when participants rated the expectation of receiving a right arm shock following the CS+R presentation compared to both the CS+L and CS- ($t_{106.118} = 22.69$, $p < 0.001$; Figure S1C).

Overall, these results indicate that participants successfully acquired threat learning, as CSs+ were rated as less pleasant than the CS-, and they correctly reported the association between each CS and laterality of expected and received shock delivery.

Corticospinal excitability, cumulative sums. A rmANOVA was performed on the last trial of the MEP cumulative sums (z-scores) with muscle (FDI, ECR) and CS (CS-, CS+L, CS+R) as within-subject factors. We observed a main effect of CS ($F_{2,52} = 14.60$, $p < 0.001$, $\eta^2_p = 0.36$, 90% CI [0.17 0.48]) and an interaction between muscle and CS ($F_{2,52} = 5.3$, $p = 0.008$, $\eta^2_p = 0.17$, 90% CI [0.03 0.30]). No main effect of muscle emerged ($F_{1,26} = 0.30$, $p = 0.588$, $\eta^2_p = 0.01$, 90% CI [0 0.14]). Planned comparisons showed that the CS+R MEPs were significantly reduced compared to the CS- for both FDI ($t_{85.51} = –5.73$, $p < 0.001$) and ECR ($t_{85.51} = –3.36$, $p = 0.001$) muscles, and compared to the CS+L, but only for the FDI muscle ($t_{85.51} = 4.65$, $p < 0.001$; Figure S2A). In contrast, there was no significant difference in MEP amplitude between CS+L and CS-, neither for FDI ($t_{85.51} = –1.08$,

p = 0.283) or ECR muscle ($t_{85.51} = –1.98$, p = 0.051). These results show a significant reduction of MEP amplitudes in anticipation of side-congruent aversive stimuli for both FDI and ECR muscles.

Skin conductance response, cumulative sums. A rmANOVA was performed with CS (CS-, CS+L, CS+R) as within-subject factor on SCR cumulative sums (z-score). We observed a main effect of CS ($F_{2,48} = 10.34$, $p < 0.001$, $\eta^2_p = 0.30$, 90% CI [0.11 0.43]). Planned comparisons showed that SCR was significantly higher for both CS+L and CS+R than CS- ($t_{48} = 3.95$, p < 0.001 and $t_{48} = 3.92$, p < 0.001, respectively), and did not differ between CSs+ ($t_{48} = –0.03$, p = 0.977; Figure S2A). Participants showed higher arousal to the CSs+ than the CS-, regardless of the laterality of the expected shock.

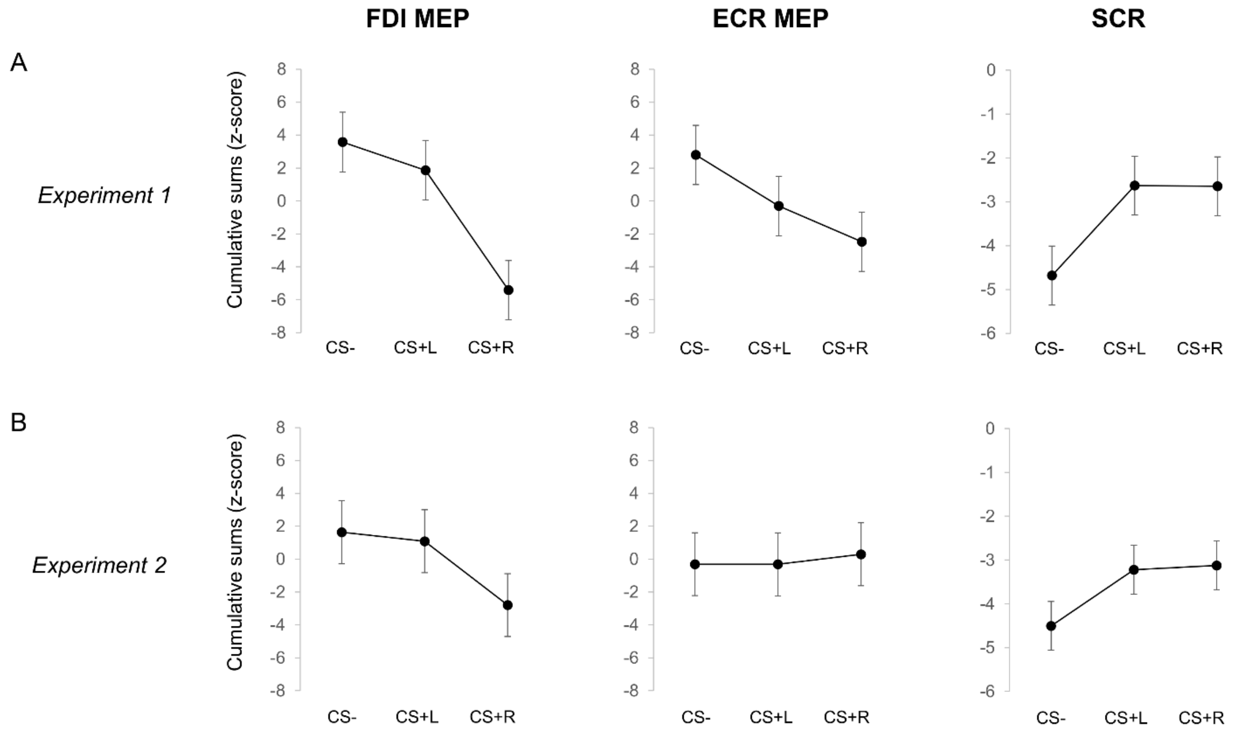

Figure S2. Corticospinal excitability and skin conductance responses cumulative sums of the last trial of threat learning. Group estimate marginal means and 95% CI of MEP amplitudes for the FDI and ECR muscles and SCR as a function of the CS type in Experiment 1 (A) and Experiment 2 (B).

Experiment 2

Self-report measures. Participants rated the CS+L and the CS+R as less pleasant than the CS- ($t_{58} = –7.65$, p < 0.001 and $t_{58} = –7.65$, p < 0.001, respectively; see FigureS1D), whereas ratings for CS+L and CS+R did not differ ($t_{58} < 0.001$, p = 1).

Regarding the expectation associated with each CS to receive a left or right US, planned contrasts on explicit subjective ratings revealed that participants rated the expectation of receiving a left arm shock when presented with a CS+L higher compared to both the CS+R and CS- ($t_{115.979} = 24.68$, p < 0.001). A similar pattern emerged when participants rated the expectation of receiving a right arm

shock following the CS+R presentation compared to both the CS+L and CS- ($t_{115.979} = 24.02$, $p < 0.001$; Figure S1E).

Similarly, planned contrasts on the reported contingency of each CS with a left or right US showed that higher scores were given when participants were to assess that they received a left arm shock when presented with a CS+L higher compared to both the CS+R and CS- ($t_{110.433} = 37.24$, $p < 0.001$). A similar pattern emerged when participants rated the expectation of receiving a right arm shock following the CS+R presentation compared to both the CS+L and CS- ($t_{110.433} = 35.59$, $p < 0.001$; Figure S1F).

As for the Experiment 1, these results overall indicate that participants successfully acquired threat learning, as CSs+ were rated as less pleasant than the CS- after the Threat learning phase, and they correctly reported the association between each CS and laterality of expected and received shock delivery.

Corticospinal excitability, cumulative sums. A rmANOVA was performed on MEP cumulative sums (z-scores) with muscle (FDI, ECR) and CS (CS-, CS+L, CS+R) as within-subject factors. We observed an interaction between muscle and CS ($F_{2,58} = 5.65$, $p = 0.006$, $\eta^2_p = 0.16$, 90% CI [0.03 0.29]). No other main effects or interaction emerged (all $F < 1.02$, $p > 0.366$). Planned comparisons showed that the CS+R MEPs were significantly reduced compared to the CS- for FDI ($t_{91.94} = 2.66$, $p = 0.009$) but not the ECR ($t_{91.94} = –0.36$, $p = 0.72$) muscle (Figure S2B). In addition, there was no significant difference in MEP amplitude between CS+L and CS-, neither for FDI ($t_{91.94} = 0.33$, $p = 0.744$) or ECR muscle ($t_{91.94} = 0.004$, $p = 0.997$). These results show a significant reduction of FDI MEP amplitude, but not of ECR muscle, in anticipation of side-congruent shock.

Skin conductance response, cumulative sums. A rmANOVA was performed on SCR cumulative sums (z-scores) with CS (CS-, CS+L, CS+R) as within-subject factor. We observed a main effect of CS ($F_{2,54} = 6.57$, $p = 0.003$, $\eta^2_p = 0.19$, 90% CI [0.05 0.32]). Planned comparisons showed that SCR was significantly higher for both CS+L and CS+R than CS- ($t_{54} = –3.01$, $p = 0.004$ and $t_{54} = –3.25$, $p = 0.002$, respectively), but did not differ between CSs+ ($t_{54} = –0.24$, $p = 0.814$; Figure S2B). Higher arousal to the CSs+ than the CS- emerged, regardless of the laterality of the expected shock.
